# Metformin administration is associated with enhanced response to transarterial chemoembolization for hepatocellular carcinoma in type 2 diabetes patients

**DOI:** 10.1038/s41598-022-18341-2

**Published:** 2022-08-25

**Authors:** Woo Jin Jung, Sangmi Jang, Won Joon Choi, Jaewon Park, Gwang Hyeon Choi, Eun Sun Jang, Sook-Hyang Jeong, Won Seok Choi, Jae Hwan Lee, Chang Jin Yoon, Jin-Wook Kim

**Affiliations:** 1 Departments of Internal Medicine, Seoul National University Bundang Hospital, Seoul National University College of Medicine, Gumi-dong 300, Seongnam, Kyonggi-Do 463-802 Republic of Korea; 2 Department of Internal Medicine, College of Medicine, Seoul National University, Seoul, Republic of Korea; 3 Department of Radiology, Seoul National University Bundang Hospital, Seoul National University College of Medicine, Seoul, Republic of Korea

**Keywords:** Hepatology, Liver diseases, Liver cancer, Hepatocellular carcinoma

## Abstract

Transarterial chemoembolization (TACE) is often used as a locoregional therapy for early hepatocellular carcinoma (HCC) when local ablation or resection are not feasible, but incomplete response and recurrence are commonly observed. In this study, we sought to determine the association between metformin administration and TACE outcomes for single nodular HCC in patients with type 2 diabetes mellitus (T2DM). The retrospective cohort analysis included 164 T2DM patients with single nodular HCC who underwent TACE as an initial treatment, and 91 were exposed to metformin before and after TACE. Propensity score (PS) matching was used to balance covariates. Logistic regression analysis was used to determine the predictors of tumor response after TACE, and Cox regression analysis assessed independent predictors of local tumor recurrence (LTR) in patients with complete response after TACE. Metformin use was associated with significantly higher objective response rate (ORR) in the overall and PS-matched cohort (79.1% vs. 60.3 and 78.7% vs. 57.5%; p = 0.008 and p = 0.029, respectively). Logistic regression analysis showed that metformin use was an independent predictor of ORR in all and PS-matched patients (odds ratio = 2.65 and 3.06; p = 0.016 and 0.034, respectively). Cox regression analysis showed metformin administration was an independent predictor for lower LTR in all and PS-matched patients (hazard ratio = 0.28 and 0.27; p = 0.001 and 0.007, respectively). Metformin administration is associated with better initial response and lower local recurrence after TACE for single nodular HCC in T2DM.

## Introduction

Hepatocellular carcinoma (HCC), the most common type of liver cancer, is the fourth leading cause of cancer death worldwide^1^. Treatment of HCC includes locoregional and systemic therapy depending on the tumor stage and the functioning hepatic reserve. Transarterial chemoembolization (TACE) is the most frequently used locoregional therapy for patients with intermediate-stage HCC^2^. In addition, early HCC may also be indicated for TACE if surgery or local ablation are not feasible^2^. However, the local control rate of TACE ranges from 57 to 80%^3–7^, and the long-term recurrence-free rate is only 17%–35% in early HCC^6,8–12^. Combined radiofrequency ablation (RFA) may enhance TACE's response rate^13–15^, but there is a concern for the risk of complications^5,11^. Therefore, it is clinically relevant to develop strategies to enhance the response rate of TACE in early HCC.

Metformin, a first-line treatment for type 2 diabetes mellitus (T2DM), has received attention as a preventive measure in several cancers, including HCC. Retrospective observational studies and meta-analyses have shown that metformin reduces the risk of developing HCC in patients with diabetes^16–18^. In addition to its preventive effect, several preclinical studies have reported that metformin may enhance the effect of cytotoxic drugs, radiofrequency ablation, or radiation therapy for HCC^19–23^. However, the effect of metformin in known HCC patients is less extensively studied. Retrospective cohort studies reported that the combination with metformin did not increase the antitumor effect of sorafenib^24,25^, and the impact of metformin on the HCC survival is controversial^26–31^. It is also not known whether metformin affects the outcomes of TACE. In this study, we aimed to assess whether metformin affects therapeutic outcomes of TACE for early HCC in patients with T2DM.

## Result

### Study population

**Figure 1:**
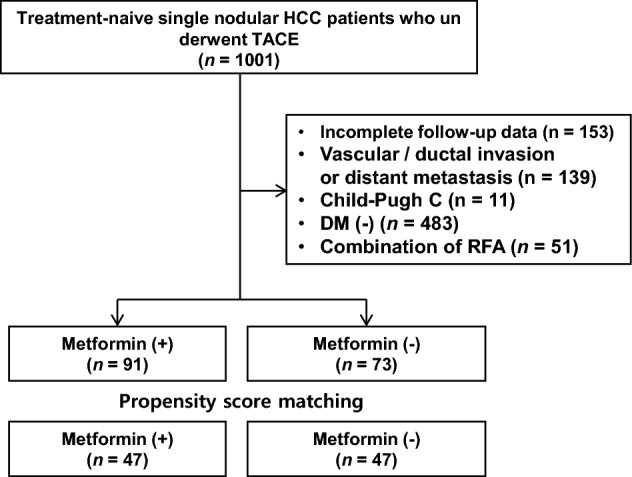
Flow chart of the study population. *HCC* hepatocellular carcinoma, *TACE* transarterial chemoembolization.

During the study period, 1,001 treatment-naïve patients with single nodular HCC underwent TACE as an initial treatment. After excluding patients with incomplete follow-up imaging data, vascular and/or ductal invasion, distant metastasis, Child–Pugh C, no diabetes or combined radiofrequency ablation, 164 patients were finally included for analysis (Fig. 1). Out of them, 91 were on metformin before TACE. After PS matching, 94 patients were selected for comparison: 47 in control and 47 in metformin group.

In the PS-matched metformin group, all patients received metformin at least for one day during the admission period for TACE, and 58 percent of patients received metformin on the day of TACE procedure. After TACE, 60 and 38 percent of patients were confirmed to maintain metformin for at least 6 months after TACE and till the end of follow-up, respectively. In the metformin group, the median duration of documented metformin therapy before TACE was 32 months (5.5 months for PS-matched patients), ranging 1–177 months (1–137 months for PS-matched patients). The median dose of metformin was 250 mg/day (range 250–1000 mg/day).

The baseline characteristics of the patients are shown in Table 1. Patients on metformin were older of age, had less frequent viral etiology of liver disease, higher rate of prescription for DPP-4 inhibitors and SGLT2 inhibitors than control. Metformin group also showed better liver function indicators (higher platelet counts, lower prothrombin time, lower bilirubin, better Albumin-Bilirubin [ALBI] grades^32^), higher BCLC staging, and larger tumor size. However, the differences between the two groups became insignificant after propensity score matching.

**Table 1:** Comparison of baseline characteristics between diabetic patients with or without metformin therapy before TACE for single nodular HCC.

Variables	Before PS matching			After PS matching		
	Control (n = 73)	Metformin (n = 91)	*p* value/SMD	Control (n = 47)	Metformin (n = 47)	*p* value/SMD
Age, years	67 (16)	71 (18)	0.073 − 0.288	67 (16)	71 (18)	0.615 − 0.104
Male gender	57 (78)	71 (78)	0.993 0.001	38 (81)	37 (79)	0.797 0.053
Heavy alcohol consumption	19 (26)	29 (32)	0.414 0.129	15 (31)	16 (33)	0.827 0.045
**Comorbidity**						
Hypertension	27 (37)	27 (30)	0.322 0.156	18 (38)	12 (25)	0.186 0.272
Cardiopulmonary disease**	9 (12)	16 (18)	0.352 0.148	7 (15)	7 (15)	1.000 0.000
CKD	12 (16)	16 (18)	0.847 0.030	8 (17)	6 (13)	0.563 0.118
Viral hepatitis	44 (60)	26 (29)	< 0.001 0.673	20 (44)	22 (49)	0.673 0.089
Liver cirrhosis	61 (84)	77 (85)	0.854 0.029	39 (81)	40 (83)	0.789 0.055
Child–Pugh class (A/B)	55/18	69/22	0.943 0.011	38/10	33/15	0.245 0.239
Child–Pugh score 5/6/7/8/9	38/17/8/5/5	52/17/11/8/3	0.756 0.215	28/10/6/2/2	26/7/6/6/3	0.591 0.347
**Other antidiabetic medications and statins**						
Sulfonylurea	19 (26)	32 (35)	0.209 0.199	15 (31)	15 (31)	1.000 0.000
Alpha glucosidase	15 (21)	13 (14)	0.290 0.166	8 (17)	8 (17)	1.000 0.000
Thiazolidinedione	6 (8)	8 (9)	0.896 0.021	3 (6)	4 (9)	0.694 0.081
DPP-4 inhibitor	5 (7)	25 (27)	0.001 0.569	27 (57)	27 (57)	1.000 0.000
SGLT2i	0 (0)	6 (7)	0.025 0.272	0 (0)	0 (0)	1.000 0.000
Insulin	64 (88)	76(83)	0.454 0.119	41 (87)	41 (87)	1.000 0.000
Statins	12 (16)	24 (26)	0.127 0.244	19 (40)	19 (40)	1.000 0.000
**Laboratory data**						
HbA1C, %	6.8 (1.7)	7.0 (2.1)	0.557 − 0.041	6.6 (1.4)	6.9 (2.0)	0.852 − 0.060
Creatinine (mg/dL)	0.96 (0.37)	0.91 (0.37)	0.351 0.276	1.00 (0.10)	0.93 (0.10)	0.329 0.330
eGFR	73 (33)	79 (33)	0.176 − 0.201	72 (40)	76 (29)	0.351 − 0.139
Platelet, × 10^3^/ul	119 (94)	135 (100)	0.010 − 0.239	130 (108)	135 (118)	0.516 − 0.108
Prothrombin time, INR	1.13 (0.18)	1.08 (0.15)	0.018 0.328/	1.11 (0.16)	1.12 (0.21)	0.553 0.078
Albumin, g/dL	3.6 (0.7)	3.9 (0.7)	0.193 − 0.194	3.7 (0.7)	3.6 (0.9)	0.315 0.184
Total bilirubin, mg/dL	0.9 (0.7)	0.7 (0.5)	0.009 0.284	0.8 (0.5)	0.8 (0.7)	0.770 − 0.054
ALBI grade^32^ 1/2/3	27/43/3	54/34/3	0.006 0.461	22/23/2	22/22/3	0.985 0.097
AFP, ng/ml	7.3 (30.1)	5.0 (23.7)	0.241 − 0.178	5.9 (22.3)	7.5 (84.5)	0.513 − 0.332
Tumor size, cm	2.7 (3.0)	3.3 (3.6)	0.026 − 0.228	3.2 (3.3)	3.3 (4.3)	0.307 − 0.201
BCLC (0/A)	25/48	17/74	0.023 0.358	14/33	10/37	0.344 0.196
DEB-TACE	5 (7)	12 (13)	0.186 0.212	5 (11)	6 (13)	0.748 0.066
Follow-up duration (months)***	22 (44)	13 (28)	0.190 0.293	46 (52)	25 (50)	0.148 0.395

*AFP* alpha-fetoprotein, *ALBI* albumin-bilirubin, *BCLC* Barcelona Clinic Liver Cancer, *CKD* chronic kidney disease, *DEB* drug-eluting bead, *DPP-4* dipeptidyl peptidase-4, *HbA1C* hemoglobin A1c (glycated), *INR* international normalized ratio, *PS* propensity score, *RFA* radiofrequency ablation, *SMD* standardized mean difference.

Categorical variables are presented as numbers (%), and tested using chi-square test. Continuous variables are presented as median (interquartile range), and p-values were calculated using Mann–Whitney *U* test.

*Heavy alcohol consumption was defined as chronic consumption of > 40 g of alcohol per day.

**Coronary heart disease, congestive heart failure and chronic obstructive pulmonary disease.

***For patients with complete response after TACE.

### Pre-TACE metformin administration as a predictor of the favorable initial response of TACE

Table 2 shows the radiological response rate of overall and PS-matched patients according to mRECIST criteria 4 weeks after initial TACE. Metformin group had a higher objective response, i.e., sum of complete response (CR) and partial response (PR), than control: 79.1% vs. 60.3%, odds ratio (OR) 2.50, p = 0.009 for all patients, and 78.7% vs. 57.5%, OR 2.74, p = 0.029 for PS-matched patients. CR was not statistically different between the two groups (OR 1.11, p = 0.738 for all patients, and OR 1.00, p = 1.000 for PS-matched patients).

**Table 2:** Initial radiological response to TACE according to metformin administration.

All patients	Control n = 73	Metformin n = 91	Odds ratio	95% CI	P value
Objective response rate (%)	44 (60.3)	72 (79.1)	2.50	1.25–4.98	0.009
Complete response (%)	39 (53.4)	51 (56.0)	1.11	0.60–2.06	0.738
Partial response (%)	5 (6.9)	21 (23.1)			
Stable disease (%)	23 (31.5)	15 (16.5)			
Progressive disease (%)	6 (8.2)	4 (4.4)			

PS-matched patients	Control n = 47	Metformin n = 47	Odds ratio	95% CI	P value
Objective response rate (%)	27 (57.5)	37 (78.7)	2.74	1.11–6.79	0.029
Complete response (%)	25 (53.2)	25 (53.2)	1.00	0.44–2.25	1.000
Partial response (%)	2 (4.2)	12 (25.5)			
Stable disease (%)	16 (34.0)	7 (14.9)			
Progressive disease (%)	4 (8.5)	3 (6.4)			

Objective response rate: complete response + partial response.

Logistic regression analysis showed that metformin use was an independent predictor of objective response, along with tumor size in both overall (OR 2.65, 95% CI 1.20–5.84, p = 0.016) and PS-matched (OR 3.06, 95% CI 1.09–8.59, p = 0.034) patients (Supplementary Tables 1 and 2). The daily dose of metformin did not have significant effect on TACE response in overall patients (OR 2.14, p = 0.174), but patients with daily metformin dose > 500 mg had higher objective response in PS-matched group (OR 6.27, p = 0.049).

### Metformin use was associated with a low incidence of local tumor recurrence after TACE

**Figure 2:**
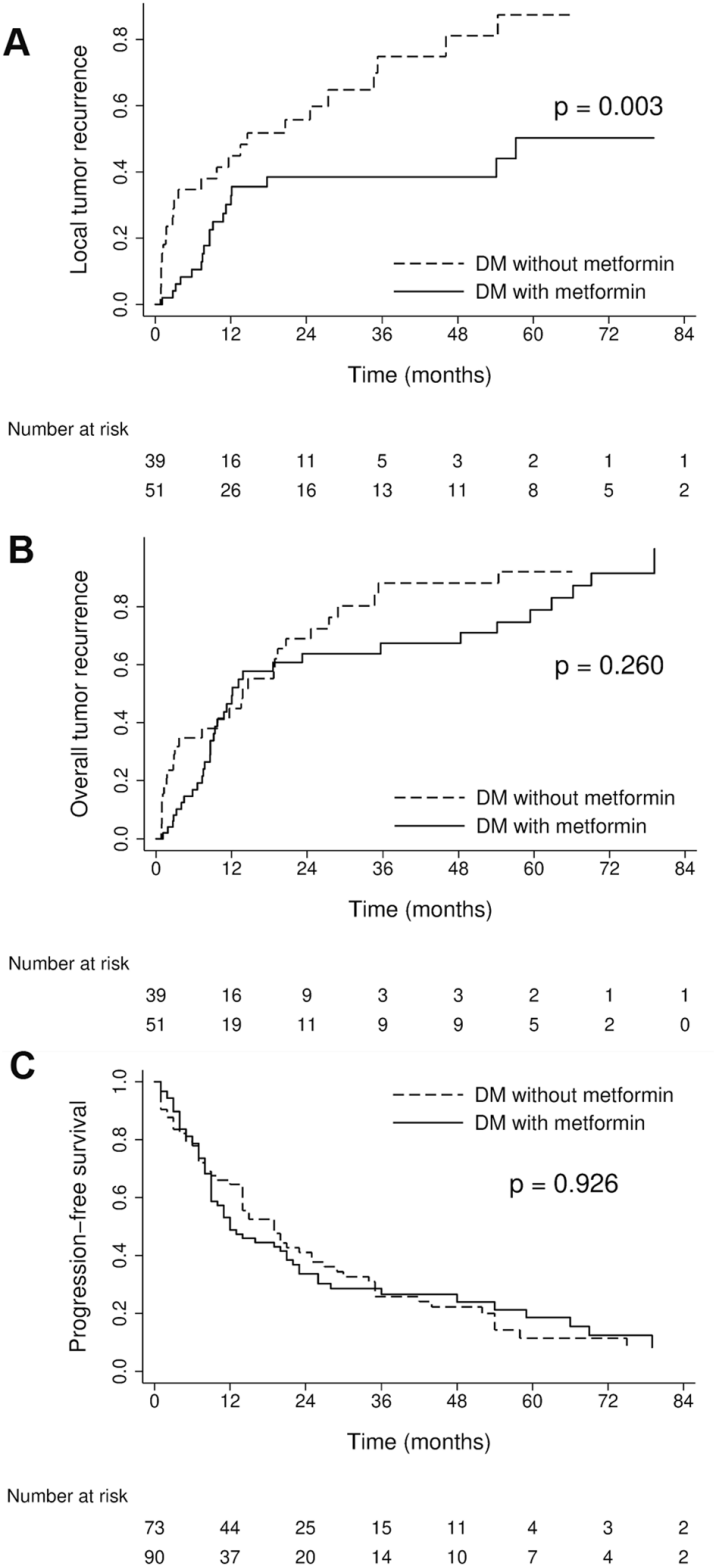
Kaplan–Meier plots for local tumor recurrence-free survival, overall recurrence-free survival and progression free survival after TACE according to metformin therapy. Local and overall recurrent-free survivals were analyzed in patients with complete response after initial TACE (**A** and **B**; n = 90), and the progression-free survival was calculated in overall patients (**C**; n = 163). The metformin group showed a significantly reduced local recurrence rate than the control group (**A**), but the overall recurrence was not different between the two groups (**B**). Progression-free survival was not different between control and metformin group, either (**C**). DM without metformin, patients with T2DM not treated with metformin; DM with metformin, patients with T2DM treated with metformin. *HCC* hepatocellular carcinoma, *DM* diabetes mellitus.

Overall, 90 out of 164 patients (54.9%) achieved CR after single session of TACE. Of these patients with CR, the overall recurrence and local recurrence rates were 70.0% and 47.8%, respectively. Kaplan–Meier analysis showed that local tumor recurrence (LTR) was significantly lower in patients taking metformin than in patients without metformin administration before TACE (p = 0.003) (Fig. 2A). However, the overall recurrence rate was not significantly different between the two groups (p = 0.260) (Fig. 2B). Progression-free survival was not different between control and metformin group (p = 0.926) (Fig. 2C), either. Multivariate Cox regression analysis showed that metformin administration was an independent predictor for LTR (hazard ratio [HR] 0.28, 95% CI 0.14–0.58, p = 0.001), along with hypertension, pre-treatment AFP levels and tumor size (Table 3). After covariates were balanced by PS matching, metformin administration was still a significant predictor of low LTR in patients with T2DM (HR 0.27, 95% confidence interval 0.11–0.70; p = 0.007, Supplementary Table 3). In metformin group, the daily dose of metformin did not have significant effect on local recurrence (OR 0.78 and 0.93, p = 0.500 and 0.873 in all and PS-matched patients, respectively).

**Table 3:** Cox regression analysis for predictors of local HCC recurrence in all diabetic patients with complete response after TACE (N = 90).

Variables	Univariate		Multivariate	
	HR (95% CI)	*P* value	HR (95% CI)	*P* value
Age (year)	0.99 (0.96–1.01)	0.346		
Male gender	0.86 (0.39–1.89)	0.714		
Heavy alcohol consumption	0.66 (0.34–1.28)	0.224		
Hypertension	0.48 (0.23–0.96)	0.039	0.40 (0.19–0.86)	0.019
Cardiopulmonary disease	1.34 (0.52–3.40)	0.544		
CKD	0.83 (0.33–2.11)	0.695		
Viral hepatitis	1.62 (0.88–2.97)	0.119		
Liver cirrhosis	2.04 (0.73–5.72)	0.176		
Child–Pugh class B over A	1.71 (0.82–3.53)	0.150		
Metformin	0.40 (0.21–0.74)	0.003	0.28 (0.14–0.58)	0.001
Metformin dose > 500 mg/day	0.78 (0.39–1.59)	0.500		
Sulfonylurea	0.76 (0.37–1.54)	0.441		
Alpha glucosidase	0.88 (0.39–1.99)	0.765		
Thiazolidinedione	1.49 (0.59–3.81)	0.401		
DPP-4 inhibitor	0.64 (0.35–1.16)	0.140		
SGLT2i	0.31 (0.04–2.23)	0.242		
Insulin	1.66 (0.59–4.65)	0.334		
Statin	0.68 (0.37–1.27)	0.226		
HbA1C	1.10 (0.92–1.34)	0.287		
eGFR	0.99 (0.98–1.00)	0.078	0.99 (0.98–1.00)	0.128
Platelet, × 10^3^/uL	1.00 (1.00–1.00)	0.532		
Prothrombin time (INR)	3.30 (0.38–28.28)	0.276		
Albumin, g/dL	0.76 (0.41–1.40)	0.378		
Total bilirubin, mg/dL	1.25 (0.79–1.98)	0.340		
ALBI grade	1.34 (0.74–2.43)	0.33303		
AFP > 20 ng/mL	2.68 (1.42–5.05)	0.001	2.18 (1.11–4.29)	0.024
Tumor size (cm)	1.22 (1.01–1.48)	0.037	1.34 (1.07–1.68)	0.011
BCLC A over 0	1.61 (0.79–3.27)	0.189		
DEB-TACE	1.38 (0.49–3.90)	0.549		

*TACE* transarterial chemoembolization, *HCC* hepatocellular carcinoma, *HR* hazard ratio, *CI* confidence interval, *AFP* alpha-fetoprotein, *INR* international normalized ratio, *RFA* radiofrequency ablation.

## Discussion

Although TACE is not recommended as a first-line treatment for early HCC, it has been performed in early HCC in real-world practice for variable reasons such as a bridge to transplantation^33^ or infeasible for local ablation and surgery^3,6–8,10,12,34^ A large-scale survey revealed that 28% of all TACE procedures were performed in early HCC, i.e., Barcelona Clinic Liver Cancer (BCLC) stage A^4^. Another global study reported that similar proportion of patients with BCLC 0/A received TACE as an initial treatment^34^. Since the long-term local control rate is relatively low in this setting^6,8–12^, it is a clinically relevant issue to enhance the response rate of TACE in BCLC 0/A. Our results also showed that only two-thirds of patients with single HCC obtained CR after TACE, and one-thirds of the patients with CR eventually experienced local tumor recurrence.

Numerous epidemiologic studies reported association between metformin use and decreased risk of certain cancers such as pancreatic cancer and colorectal cancer in patients with T2DM^35^. In terms of HCC, meta-analyses and large-scale cohort studies have shown that metformin administration is associated with a reduced risk of HCC^16–18^. Controversy still exists, however, on the association between cancer risks and metformin^36^. It is even less certain whether metformin has an adjuvant effect in cancer therapy^37^. Anecdotal studies have reported favorable effects of metformin after resection, radiotherapy, and ablation for HCC^30,31,38–40^. However, it has not yet been reported whether metformin affects the outcomes of TACE for HCC. In this study, we found that exposure to metformin was associated with favorable outcome when TACE was chosen as an initial therapy for single HCC. The beneficial effect of metformin remained significant after controlling potential confounders such as liver function, stage of HCC, comorbidities and use of other antidiabetic drugs by PS matching.

Metformin activates 5′-AMP-activated protein kinase (AMPK), which in turn may suppress tumor cell proliferation and metabolism^41,42^. However, metformin may also antagonize cisplatin-induced cytotoxicity in certain cancer cells^42^. The biological mechanisms underlying our observations are not certain at the present time. TACE induces HCC cell death by increasing the local concentration of cytotoxic drugs and causing ischemia^43^. However, TACE-induced ischemia may also lead to neo-angiogenesis of residual HCC via increased local HIF-1a and vascular endothelial growth factor concentrations^44^. Furthermore, hypoxia may confer resistance to chemotherapeutics against HCC cells^45^. Preclinical studies have suggested that metformin inhibits tumor angiogenesis by inhibiting the HIF-1a and VEGF-signaling pathways^46,47^. Metformin may also attenuate hypoxia-induced resistance to chemotherapeutics in the hepatoma cells^48^. Thus, we speculate that metformin creates favorable environment for TACE-induced cell death in the liver, leading to enhanced tumor response to TACE and subsequently less frequent local relapse. This hypothesis may explain the finding that metformin use was associated with a lower risk of local recurrence but overall recurrence and PFS were not affected, possibly due to the high incidence of metachronous multiple HCC in our patients. Consecutive ablation therapy may also have achieved further local HCC control in patients without CR after initial TACE, reducing the potential beneficial effect of metformin in terms of PFS. We do not believe that metformin per se has sufficient antitumor effect to suppresses intrahepatic metastasis or multicentric HCC, because HCC developed while many of our patients were on metformin. However, the exact mechanism of action of metformin on TACE response needs to be explored in further studies.

Our multivariate model showed that tumor size was an additional independent predictor for initial TACE response and local recurrence. Size of HCC has repeatedly been reported to predict aggressive tumor behavior and local control failure after TACE^7–9,12^. Although TACE may have favorable outcome comparable to local ablation therapy as an initial treatment for single nodular HCC^3^ and a recent guideline recommends TACE for early HCC in selected cases^2^, our data reconfirm that TACE may not be sufficient for local control of early HCC with large tumor size. We believe our study may provide useful background for developing a new strategy on locoregional therapy for early HCC.

Our patients received metformin for variable duration and with different doses. Multivariate analyses showed significant association between daily dose of metformin and TACE responses only in PS-matched patients, and it is clear whether metformin doses may have significant impact. It is also not certain whether maintenance of metformin other than peri-TACE dosage may affect the local recurrence. Optimal scheduling of metformin may need to be explored by controlled trials.

The present study had other limitations. Since this was a retrospective single-center study with limited number of patients in each group enrolled over a wide time interval, further validation is warranted in larger number of patients prospectively. Second, we do not have data regarding the reason(s) why control patients did not receive metformin, currently first line therapy, because choice of antidiabetic drugs may potentially introduce selection bias. It is our speculation that some patients may have switched over to insulin, and other many have started and modified oral antidiabetic drugs other than metformin long before metformin has become the first-line therapy. To minimize selection bias, we balanced the use of other antidiabetic drugs by PS matching. Third, the initial response and LTR were investigated as surrogate markers of the metformin effect on the TACE anti-tumor efficacy rather than overall survival. However, survival may be more dependent on subsequent therapies against local and/or distant HCC recurrence^49^. Lastly, our analysis was limited to early HCC, and it is unknown whether metformin would augment the efficacy of TACE in more advanced disease, i.e., BCLC-B for whom TACE is recommended as a first-line therapy. This clinically important issue may be addressed by further prospective trials.

In summary, metformin administration was independently associated with an increased initial response after TACE for single HCC in patients with T2DM. Moreover, patients who achieved CR after TACE showed lower local recurrence when exposed to metformin.

## Methods

### Study population and data collection

This retrospective cohort analysis included patients with Barcelona Clinic Liver Cancer (BCLC) stage 0/A single nodular HCC who underwent TACE as an initial treatment between April 2003 and February 2020 in a tertiary referral hospital in South Korea. Demographic, clinical, and laboratory data were retrieved using our hospital’s clinical data warehouse package of the electronic medical record system^50^. The study protocol was approved by Seoul National University Bundang Hospital Institutional Review Board (IRB No. B-2006/618-107), in accordance with the Declaration of Helsinki Ethical Principles for Medical Research involving human subjects. Seoul National University Bundang Hospital Institutional Review Board also waived informed consent due to the retrospective nature of this study and minimal expected risk to subjects.

History of all prescribed medications were retrieved by using electronic medical record system of our institution^51^. Use of metformin and other medications listed in Table 1 was defined as presence of corresponding prescription(s) during the in-hospital admission period for the TACE procedure, which typically took 3–5 days. Patients who stopped metformin before TACE were classified as control, and patients who initiated metformin therapy after TACE were excluded from analysis.

Diagnosis of HCC was confirmed based on histopathology or radiologic criteria^52,53^. Patients were excluded from the study if they had (1) incomplete imaging study, (2) vascular or ductal invasion or distant metastasis, and (3) Child–Pugh class C cirrhosis. Liver cirrhosis was diagnosed histologically or clinically as previously reported: ultrasound features of cirrhosis (coarse liver echotexture with nodularity) plus evidence of portal hypertension including ascites, splenomegaly, thrombocytopenia (< 100 × 10^9^/L) and varices^54^. T2DM was diagnosed following the American Diabetes Association guidelines^55^. Information about alcohol consumption was retrieved from the medical records. Attending hepatologists (GH Choi, ES Jang, SH Jeong and JW Kim) assessed the history of alcohol use by using structured fields of status, types of alcohol, amount of alcohol/week. Heavy alcohol consumption was defined as chronic consumption of > 40 g of alcohol per day^56^.

### Treatment selection

The recommendation for treatment selection of single nodular HCC was made by a multidisciplinary tumor board which was comprised of hepatologists, surgeons, and interventional radiologists, in accordance with the general principles of current guidelines^52,57^. TACE had been performed for early (BCLC 0/A) HCC in this study for various reasons: initial misclassification (BCLC B), patients’ preference for non-invasive procedure, or tumor location not feasible for complete ablation. Combined RFA and TACE was performed if RFA was planned for single nodular HCC but the tumor nodule could not be localized by ultrasound: lipiodol-drug emulsion was visualized for targeting either by ultrasound or cone beam CT.

### TACE procedures

A 5-F angiographic catheter was introduced (RH; Cook, Bloomington, Indiana) to the right femoral artery and visceral arteriograms were obtained to select the tumor-feeding vessels. A 3-F (Renegade, Boston Scientific, Natick, MA) or 2-F (Progreat, Terumo, Tokyo, Japan) microcatheter was used to super-select the tumor-feeding arteries. After placement of the microcatheter tip in the proper feeders, emulsion of 10–50 mg of doxorubicin hydrochloride (Adriamycin RDF; Ildong Pharmaceutical, Seoul, Korea) solution in non-ionic contrast media mixed with 2–10 mL of iodized oil (Lipiodol Ultra Fluid; Andre Guerbet, Aulnay-sous-Bois, France) in a 1:4 volume ratio was administered, and the tumor-feeding arteries were subsequently embolized with gelatin sponge particles (Gelfoam; Upjohn, Kalamazoo, MI). Cone beam CT was routinely used to assess the effectiveness of embolization. For drug-eluting bead (DEB)-TACE, 50–75 mg of doxorubicin was loaded into one vial containing 2 mL of microspheres (DC Bead, BTG) for 2 h and the preparation was suspended in 50 mL of a mixture of normal saline and contrast agent at a 1:1 ratio. One or two vials of DEB agent of 100–300 or 300–500 μm were used. DEB particles were slowly infused through tumor-feeding arteries until near stasis of arterial flow. The choice of conventional vs. DEB-TACE was on the discretion of attending physicians, taking patients’ preference into consideration.

### Assessment of treatment response

Liver four-phase dynamic computed tomography (CT) or magnetic resonance (MR) images were obtained at baseline, and CT was followed 1 month after TACE for the assessment of TACE response. Thereafter, CT or MR imaging was obtained at 3–6 months of interval. The target lesion's treatment response was assessed according to the modified Response Evaluation Criteria in Solid Tumors; complete response (CR) was defined as the disappearance of any intratumoral arterial enhancement in the target lesion. Partial response (PR) was defined as > 30% decrease in the sum of the longest viable tumor diameters of the target lesion^58,59^. The objective response rate (ORR) was defined as the combined CR and PR rate of the target lesion only, excluding new lesions' assessment. The radiologic response was assessed either by four radiologists of more than 15 years of experience in our institution, or by one of the authors (WJJ) who were not aware of the history of metformin exposure at the time of imaging analysis.

Local tumor recurrence (LTR) in patients with CR after TACE was defined as the appearance of new tumor foci at the embolized and/or ablative tumor margins^60^. Overall recurrence was defined as presence of either LTR and/or intrahepatic distant recurrence. LTR-free survival was defined as the interval between the initial TACE and the first evidence of LTR. Overall recurrence-free survival was defined as the interval between initial TACE and the first evidence of overall recurrence. Progression-free survival (PFS) was defined as the interval between TACE and the first evidence of HCC progression (local tumor progression, intrahepatic distant recurrence, gross vascular invasion, or extrahepatic distant metastasis)^61^. Overall survival was not assessed due to lack of complete survival data.

### Statistical analysis

Continuous variables were tested using the Student’s t-test or Mann–Whitney test, and categorical variables were tested using the Chi-square test. Kaplan–Meier plots and log-rank tests were used to assess LTR, overall recurrence and PFS. Logistic regression analysis was used to determine the predictors of the objective response after TACE. In patients with CR after TACE, Cox regression analysis was used to assess the independent predictors of LTR and overall recurrence. All variables with univariate regression p value < 0.1 were included in multivariate regression analysis. Subgroup analyses were conducted on patients with T2DM with CR after TACE. Propensity score (PS) matching was performed to balance the following variables between diabetic patients with and without metformin: age, chronic viral hepatitis, liver cirrhosis, use of antidiabetic drugs (sulfonylurea, DPP-4 inhibitors, SGLT2 inhibitors and insulins) and statins, platelet counts, prothrombin time, ALBI-score, pre-treatment HCC size. PS was calculated with complete case analysis and logistic regression model by using MatchIt R package (version 4.1.0). One-to-one matching was performed with nearest neighbor matching algorithm and caliper of 0.2 of the standard deviation of the estimated propensity scores. Balancing diagnostics were assessed by both p value and standardized mean difference (SMD). Statistical analysis was performed using STATA ver. 14.2 or R package ver. 4.0.2.

## Supplementary Information

Supplementary Tables.

## References

[1] Bray F (2018). Global cancer statistics 2018: GLOBOCAN estimates of incidence and mortality worldwide for 36 cancers in 185 countries. CA Cancer J. Clin..

[2] Reig M (2022). BCLC strategy for prognosis prediction and treatment recommendation: The 2022 update. J. Hepatol..

[3] Yang HJ (2014). Small single-nodule hepatocellular carcinoma: Comparison of transarterial chemoembolization, radiofrequency ablation, and hepatic resection by using inverse probability weighting. Radiology.

[4] Bargellini I (2012). Transarterial chemoembolization in very early and early-stage hepatocellular carcinoma patients excluded from curative treatment: A prospective cohort study. Eur. J. Radiol..

[5] Hyun D (2016). Early stage hepatocellular carcinomas not feasible for ultrasound-guided radiofrequency ablation: Comparison of transarterial chemoembolization alone and combined therapy with transarterial chemoembolization and radiofrequency ablation. Cardiovasc. Intervent. Radiol..

[6] Yun BY (2020). Prognosis of early-stage hepatocellular carcinoma: Comparison between trans-arterial chemoembolization and radiofrequency ablation. Cancers.

[7] Baek MY (2019). Clinical outcomes of patients with a single hepatocellular carcinoma less than 5 cm treated with transarterial chemoembolization. Korean J. Intern. Med..

[8] Hsu KF (2012). Superselective transarterial chemoembolization vs hepatic resection for resectable early-stage hepatocellular carcinoma in patients with Child-Pugh class a liver function. Eur. J. Radiol..

[9] Kim JH (2017). The role of scheduled second TACE in early-stage hepatocellular carcinoma with complete response to initial TACE. Clin. Mol. Hepatol..

[10] Song YG (2015). Transarterial chemoembolization as first-line therapy for hepatocellular carcinomas infeasible for ultrasound-guided radiofrequency ablation: A retrospective cohort study of 116 patients. Acta Radiol..

[11] Kim W (2019). Combination therapy of transarterial chemoembolization (TACE) and radiofrequency ablation (RFA) for small hepatocellular carcinoma: Comparison with TACE or RFA monotherapy. Abdom. Radiol..

[12] Terzi E (2014). TACE performed in patients with a single nodule of hepatocellular carcinoma. BMC Cancer.

[13] Peng ZW (2013). Radiofrequency ablation with or without transcatheter arterial chemoembolization in the treatment of hepatocellular carcinoma: A prospective randomized trial. J. Clin. Oncol..

[14] Ren Y (2019). Improved clinical outcome using transarterial chemoembolization combined with radiofrequency ablation for patients in Barcelona clinic liver cancer stage A or B hepatocellular carcinoma regardless of tumor size: Results of a single-center retrospective case control study. BMC Cancer.

[15] Zhang YJ, Chen MS, Chen Y, Lau WY, Peng Z (2021). Long-term outcomes of transcatheter arterial chemoembolization combined with radiofrequency ablation as an initial treatment for early-stage hepatocellular carcinoma. JAMA Netw. Open.

[16] Singh S, Singh PP, Singh AG, Murad MH, Sanchez W (2013). Anti-diabetic medications and the risk of hepatocellular cancer: A systematic review and meta-analysis. Am. J. Gastroenterol..

[17] Ma S, Zheng Y, Xiao Y, Zhou P, Tan H (2017). Meta-analysis of studies using metformin as a reducer for liver cancer risk in diabetic patients. Medicine.

[18] Tseng CH (2018). Metformin and risk of hepatocellular carcinoma in patients with type 2 diabetes. Liver Int..

[19] Petrushev B (2012). Metformin plus PIAF combination chemotherapy for hepatocellular carcinoma. Exp. Oncol..

[20] Guo Z (2016). Metformin inhibits the prometastatic effect of sorafenib in hepatocellular carcinoma by upregulating the expression of TIP30. Cancer Sci..

[21] Ling S (2017). Combination of metformin and sorafenib suppresses proliferation and induces autophagy of hepatocellular carcinoma via targeting the mTOR pathway. Int. J. Oncol..

[22] Zhang Q, Kong J, Dong S, Xu W, Sun W (2017). Metformin exhibits the anti-proliferation and anti-invasion effects in hepatocellular carcinoma cells after insufficient radiofrequency ablation. Cancer Cell. Int..

[23] Zhang KF, Wang J, Guo J, Huang YY, Huang TR (2019). Metformin enhances radiosensitivity in hepatocellular carcinoma by inhibition of specificity protein 1 and epithelial-to-mesenchymal transition. J. Cancer Res. Ther..

[24] CasadeiGardini A (2017). Metformin and insulin impact on clinical outcome in patients with advanced hepatocellular carcinoma receiving sorafenib: Validation study and biological rationale. Eur. J. Cancer.

[25] Chung YK (2018). Absence of antitumor effects of metformin in sorafenib-treated patients with hepatocellular carcinoma recurrence after hepatic resection and liver transplantation. Ann. Hepatobiliary Pancreat. Surg..

[26] Ma SJ, Zheng YX, Zhou PC, Xiao YN, Tan HZ (2016). Metformin use improves survival of diabetic liver cancer patients: Systematic review and meta-analysis. Oncotarget.

[27] Schulte L (2019). Treatment with metformin is associated with a prolonged survival in patients with hepatocellular carcinoma. Liver Int..

[28] Zhou J (2019). Meta-analysis: The efficacy of metformin and other anti-hyperglycemic agents in prolonging the survival of hepatocellular carcinoma patients with type 2 diabetes. Ann. Hepatol..

[29] Bhat M (2014). Metformin does not improve survival in patients with hepatocellular carcinoma. World J. Gastroenterol..

[30] Seo YS (2016). Association of metformin use with cancer-specific mortality in hepatocellular carcinoma after curative resection: A nationwide population-based study. Medicine.

[31] Jang WI (2015). Survival advantage associated with metformin usage in hepatocellular carcinoma patients receiving radiotherapy: A propensity score matching analysis. Anticancer Res..

[32] Johnson PJ (2015). Assessment of liver function in patients with hepatocellular carcinoma: A new evidence-based approach-the ALBI grade. J. Clin. Oncol..

[33] Hodavance MS (2016). Effectiveness of transarterial embolization of hepatocellular carcinoma as a bridge to transplantation. J. Vasc. Interv. Radiol..

[34] Park JW (2015). Global patterns of hepatocellular carcinoma management from diagnosis to death: The BRIDGE Study. Liver Int..

[35] Yu H (2019). The potential effect of metformin on cancer: An umbrella review. Front. Endocrinol..

[36] Suissa S, Azoulay L (2014). Metformin and cancer: Mounting evidence against an association. Diabetes Care.

[37] Coyle C, Cafferty FH, Vale C, Langley RE (2016). Metformin as an adjuvant treatment for cancer: A systematic review and meta-analysis. Ann. Oncol..

[38] Chan KM (2017). Metformin confers risk reduction for developing hepatocellular carcinoma recurrence after liver resection. Liver Int..

[39] Chen TM, Lin CC, Huang PT, Wen CF (2011). Metformin associated with lower mortality in diabetic patients with early stage hepatocellular carcinoma after radiofrequency ablation. J. Gastroenterol. Hepatol..

[40] Shen C (2016). Sirolimus and metformin synergistically inhibit hepatocellular carcinoma cell proliferation and improve long-term survival in patients with HCC related to hepatitis B virus induced cirrhosis after liver transplantation. Oncotarget.

[41] Saengboonmee C, Sanlung T, Wongkham S (2021). Repurposing metformin for cancer treatment: A great challenge of a promising drug. Anticancer Res..

[42] Pernicova I, Korbonits M (2014). Metformin-mode of action and clinical implications for diabetes and cancer. Nat. Rev. Endocrinol..

[43] Lencioni R (2010). Loco-regional treatment of hepatocellular carcinoma. Hepatology.

[44] Liu K (2016). The changes of HIF-1alpha and VEGF expression after TACE in patients with hepatocellular carcinoma. J. Clin. Med. Res..

[45] Zhou QY (2017). GC7 blocks epithelial-mesenchymal transition and reverses hypoxia-induced chemotherapy resistance in hepatocellular carcinoma cells. Am. J. Transl. Res..

[46] Zhou X (2016). Metformin suppresses hypoxia-induced stabilization of HIF-1alpha through reprogramming of oxygen metabolism in hepatocellular carcinoma. Oncotarget.

[47] Wang J (2015). Suppression of tumor angiogenesis by metformin treatment via a mechanism linked to targeting of HER2/HIF-1alpha/VEGF secretion axis. Oncotarget.

[48] Fujita H (2019). Metformin attenuates hypoxia-induced resistance to cisplatin in the HepG2 cell line. Oncol. Lett..

[49] Moon H (2017). All-treatment array of hepatocellular carcinoma from initial diagnosis to death: Observation of cumulative treatments. J. Cancer Res. Clin. Oncol..

[50] Yoo S (2012). Seoul National University Bundang Hospital's electronic system for total care. Healthc. Inf. Res..

[51] Yoo S, Hwang H, Jheon S (2016). Hospital information systems: Experience at the fully digitized Seoul National University Bundang Hospital. J. Thorac. Dis..

[52] European Association for the Study of the Liver (2018). EASL clinical practice guidelines: Management of hepatocellular carcinoma. J. Hepatol..

[53] Heimbach JK (2018). AASLD guidelines for the treatment of hepatocellular carcinoma. Hepatology.

[54] Lee CS (2018). Liver volume-based prediction model stratifies risks for hepatocellular carcinoma in chronic hepatitis B patients on surveillance. PLoS ONE.

[55] American Diabetes Association (2018). Classification and diagnosis of diabetes: Standards of medical care in diabetes-2018. Diabetes Care.

[56] Seitz HK (2018). Alcoholic liver disease. Nat. Rev. Dis. Primers.

[57] Marrero JA (2018). Diagnosis, staging, and management of hepatocellular carcinoma: 2018 Practice guidance by the American Association for the study of liver diseases. Hepatology.

[58] Llovet JM, Lencioni R (2020). mRECIST for HCC: Performance and novel refinements. J. Hepatol..

[59] Eisenhauer EA (2009). New response evaluation criteria in solid tumours: Revised RECIST guideline (version 1.1). Eur. J. Cancer.

[60] Ahmed M (2014). Image-guided tumor ablation: Standardization of terminology and reporting criteria: A 10-year update. Radiology.

[61] Park C (2021). Imaging predictors of survival in patients with single small hepatocellular carcinoma treated with transarterial chemoembolization. Korean J. Radiol..

