# Supplementary material for: Metformin administration is associated with enhanced response to transarterial chemoembolization for hepatocellular carcinoma in type 2 diabetes patients
Source: Sci Rep. 2022 Aug 25;12:14482. doi: 10.1038/s41598-022-18341-2 (PMC9411109; doi:10.1038/s41598-022-18341-2)
Supplement: Supplementary file 1 — Supplementary Tables. [file 41598_2022_18341_MOESM1_ESM.docx]

**Supplementary tables**

**Supplementary Table 1. Logistic regression model for predictors of objective response after TACE in all diabetic patients with single nodular HCC (n = 164)**

|  | **Univariate** | | **Multivariate** | |
| --- | --- | --- | --- | --- |
| **Variables** | **OR (95% CI)** | ***P* value** | **OR (95% CI)** | ***p* value** |
| Age (yr) | 0.98 (0.95-1.00) | 0.120 |  |  |
| Male gender | 1.32 (0.63-2.76) | 0.459 |  |  |
| Heavy alcohol consumption | 1.21 (0.60-2.42) | 0.595 |  |  |
| Hypertension | 0.60 (0.31-1.15) | 0.122 |  |  |
| Cardiopulmonary disease | 0.64 (0.28-1.46) | 0.291 |  |  |
| CKD | 0.93 (0.39-2.23) | 0.872 |  |  |
| Viral hepatitis | 1.41 (0.75-2.64) | 0.284 |  |  |
| Liver cirrhosis | 0.97 (0.39-2.41) | 0.945 |  |  |
| Child-Pugh class B over A | 0.88 (0.42-1.82) | 0.728 |  |  |
| Metformin | 2.50 (1.25-4.98) | 0.009 | 2.65 (1.20-5.84) | 0.016 |
| Metformin dose > 500 mg/d | 2.33 (0.90-6.05) | 0.081 | 2.14 (0.71-6.41) | 0.174 |
| Sulfonylurea | 1.15 (0.59-2.22) | 0.679 |  |  |
| Alpha glucosidase | 1.16 (0.51-2.62) | 0.727 |  |  |
| Thiazolidinedione | 0.86 (0.32-2.32) | 0.763 |  |  |
| DPP-4 inhibitor | 1.49 (0.61-3.63) | 0.376 |  |  |
| SGLT2i | 2.35 (0.28-19.54) | 0.430 |  |  |
| Insulin | 1.18 (0.63-2.21) | 0.596 |  |  |
| Statin | 0.56 (0.27-1.16) | 0.120 |  |  |
| HbA1C | 1.09 (0.88-1.34) | 0.416 |  |  |
| eGFR | 2.29 (1.00-1.03) | 0.022 | 1.01 (0.99-1.02) | 0.124 |
| Platelet, x103/ul | 0.99 (0.99-1.00) | 0.045 | 0.99 (0.99-1.00) | 0.534 |
| Prothrombin time (INR) | 2.24 (0.26-19.41) | 0.463 |  |  |
| Albumin, g/dL | 1.50 (0.83-2.72) | 0.184 |  |  |
| Total bilirubin, mg/dL | 1.05 (0.64-1.72) | 0.838 |  |  |
| ALBI grade | 0.75 (0.43-1.30) | 0.301 |  |  |
| AFP > 20 ng/ml | 0.49 (0.24-0.99) | 0.049 | 0.65 (0.29-1.45) | 0.294 |
| Tumor size (cm) | 0.86 (0.78-0.95) | 0.003 | 0.86 (0.76-0.97) | 0.016 |
| BCLC A over 0 | 0.80 (0.42-1.55) | 0.514 |  |  |
| DEB-TACE | 0.77 (0.26-2.29) | 0.636 |  |  |
| TACE, transarterial chemoembolization; HCC, hepatocellular carcinoma; HR, hazard ratio; CI, confidence interval; AFP, alpha-fetoprotein; INR, international normalized ratio; RFA, radiofrequency ablation. | | | | |

**Supplementary Table 2. Logistic regression model for predictors of objective response after TACE in PS-matched diabetic patients with single nodular HCC (n = 94)**

|  | **Univariate** | | **Multivariate** | |
| --- | --- | --- | --- | --- |
| **Variables** | **OR (95% CI)** | ***P* value** | **OR (95% CI)** | ***p* value** |
| Age (yr) | 0.98 (0.95-1.00) | 0.120 |  |  |
| Male gender | 1.32 (0.63-2.76) | 0.459 |  |  |
| Heavy alcohol consumption | 1.21 (0.60-2.42) | 0.595 |  |  |
| Hypertension | 0.60 (0.31-1.15) | 0.122 |  |  |
| Cardiopulmonary disease | 0.64 (0.28-1.46) | 0.291 |  |  |
| CKD | 0.93 (0.39-2.23) | 0.872 |  |  |
| Viral hepatitis | 1.41 (0.75-2.64) | 0.284 |  |  |
| Liver cirrhosis | 0.97 (0.39-2.41) | 0.945 |  |  |
| Child-Pugh class B over A | 0.88 (0.42-1.82) | 0.728 |  |  |
| Metformin | 2.74 (1.11-6.79) | 0.029 | 3.06 (1.09-8.59) | 0.034 |
| Metformin dose > 500 mg/d | 4.67 (1.00-21.81) | 0.050 | 6.27 (1.01-38.93) | 0.049 |
| Sulfonylurea | 1.15 (0.59-2.22) | 0.679 |  |  |
| Alpha glucosidase | 1.16 (0.51-2.62) | 0.727 |  |  |
| Thiazolidinedione | 0.86 (0.32-2.32) | 0.763 |  |  |
| DPP-4 inhibitor | 1.49 (0.61-3.63) | 0.376 |  |  |
| SGLT2i | 2.35 (0.28-19.54) | 0.430 |  |  |
| Insulin | 1.18 (0.63-2.21) | 0.596 |  |  |
| Statin | 0.56 (0.27-1.16) | 0.120 |  |  |
| HbA1C | 1.09 (0.88-1.34) | 0.416 |  |  |
| eGFR | 1.01 (1.00-1.03) | 0.182 |  |  |
| Platelet, x10^3^/ul | 0.99 (0.99-1.00) | 0.027 | 1.00 (0.99-1.00) | 0.244 |
| Prothrombin time (INR) | 2.24 (0.26-19.41) | 0.463 |  |  |
| Albumin, g/dL | 1.50 (0.83-2.72) | 0.184 |  |  |
| Total bilirubin, mg/dL | 1.05 (0.64-1.72) | 0.838 |  |  |
| ALBI grade | 0.75 (0.43-1.30) | 0.301 |  |  |
| AFP > 20 ng/ml | 0.50 (0.20-1.26) | 0.141 |  |  |
| Tumor size (cm) | 0.84 (0.73-0.96) | 0.011 | 0.80 (0.67-0.95) | 0.012 |
| BCLC A over 0 | 1.09 (0.41-2.93) | 0.863 |  |  |
| DEB-TACE | 0.84 (0.73-0.29) | 0.636 |  |  |
| TACE, transarterial chemoembolization; HCC, hepatocellular carcinoma; HR, hazard ratio; CI, confidence interval; AFP, alpha-fetoprotein; INR, international normalized ratio; RFA, radiofrequency ablation. | | | | |

**Supplementary Table 3. Cox regression analysis for predictors of local HCC recurrence in PS-matched diabetic patients with complete response after TACE (N =50)**

|  | **Univariate** | | **Multivariate** | |
| --- | --- | --- | --- | --- |
| **Variables** | **HR (95% CI)** | ***P* value** | **HR (95% CI)** | ***p* value** |
| Age (yr) | 0.99 (0.95-1.02) | 0.572 |  |  |
| Male gender | 0.47 (0.18-1.25) | 0.130 |  |  |
| Heavy alcohol consumption | 0.69 (0.30-1.59) | 0.382 |  |  |
| Hypertension | 0.47 (0.19-1.17) | 0.105 |  |  |
| Cardiopulmonary disease | 0.90 (0.21-3.83) | 0.885 |  |  |
| CKD | 0.45 (0.10-1.90) | 0.275 |  |  |
| Viral hepatitis | 1.48 (0.66-3.34) | 0.345 |  |  |
| Liver cirrhosis | 1.21 (0.41-3.53) | 0.732 |  |  |
| Child-Pugh class B over A | 1.43 (0.47-4.28) | 0.527 |  |  |
| Metformin | 0.45 (0.20-1.05) | 0.064 | 0.27 (0.11-0.70) | 0.007 |
| Metformin dose >500mg/d | 0.93 (0.37-2.34) | 0.873 |  |  |
| Sulfonylurea | 0.54 (0.18-1.59) | 0.263 |  |  |
| Alpha glucosidase | 0.90 (0.33-2.42) | 0.832 |  |  |
| Thiazolidinedione | 0.90 (0.33-2.42) | 0.832 |  |  |
| DPP-4 inhibitor | 0.79 (0.35-1.78) | 0.569 |  |  |
| SGLT2i | - | - |  |  |
| Insulin | 1.01 (0.30-3.39) | 0.991 |  |  |
| Statin | 0.75 (0.32-1.77) | 0.517 |  |  |
| HbA1C | 0.98 (0.72-1.33) | 0.883 |  |  |
| eGFR | 0.99 (0.97-1.01) | 0.183 |  |  |
| Platelet, x10^3^/ul | 1.00 (1.00-1.00) | 0.460 |  |  |
| Prothrombin time (INR) | 2.75 (0.20-37.82) | 0.449 |  |  |
| Albumin, g/dL | 0.88 (0.37-2.07) | 0.766 |  |  |
| Total bilirubin, mg/dL | 1.47 (0.85-2.55) | 0.172 |  |  |
| ALBI grade | 1.35 (0.60-3.05) | 0.471 |  |  |
| AFP > 20 ng/ml | 1.71 (0.71-4.16) | 0.234 |  |  |
| Tumor size (cm) | 1.38 (1.03-1.85) | 0.031 | 1.63 (1.19-2.25) | 0.003 |
| BCLC A over 0 | 1.46 (0.58-3.79) | 0.420 |  |  |
| DEB-TACE | 1.42 (0.33-6.19) | 0.637 |  |  |
| TACE, transarterial chemoembolization; HCC, hepatocellular carcinoma; HR, hazard ratio; CI, confidence interval; AFP, alpha-fetoprotein; INR, international normalized ratio; RFA, radiofrequency ablation. | | | | |
